# The poor outcome of second primary oral squamous cell carcinoma is attributed to Bmi1 upregulation

**DOI:** 10.1002/cam4.1348

**Published:** 2018-02-26

**Authors:** Qinchao Hu, Tong Wu, Xiaobing Chen, Huan Li, Zhicheng Du, Yuantao Hao, Jianmin Peng, Shanshan Tai, Ming Song, Bin Cheng

**Affiliations:** ^1^ Department of Oral Medicine Hospital of Stomatology Sun Yat‐sen University Guangzhou China; ^2^ Guangdong Provincial Key Laboratory of Stomatology Guanghua School of Stomatology Sun Yat‐sen University Guangzhou China; ^3^ Department of Intensive Care Unit Sun Yat‐sen University Cancer Center Guangzhou China; ^4^ State Key Laboratory of Oncology in South China Collaborative Innovation Center of Cancer Medicine Guangzhou China; ^5^ Department of Medical Statistics and Epidemiology School of Public Health Sun Yat‐sen University Guangzhou China; ^6^ Department of Head and Neck Surgery Sun Yat‐sen University Cancer Center Guangzhou China

**Keywords:** Bmi1, nasopharyngeal carcinoma, oral squamous cell carcinoma, prognosis, radiotherapy, second primary tumor

## Abstract

Radiotherapy for nasopharyngeal carcinoma has been reported to cause second primary oral squamous cell carcinoma (s‐OSCC). The prognosis and pathologic characteristic of s‐OSCC are largely unknown. Bmi1 was associated with the repair of radiation‐induced DNA damage, suggesting its possible involvement in the pathologic process of s‐OSCC. Herein, we compared the prognosis between s‐OSCC and primary OSCC (p‐OSCC) and explored the involvement of Bmi1 in s‐OSCC development. In this retrospective study, s‐OSCC and p‐OSCC patients were matched by propensity scores. Their outcomes were compared by univariate and multivariate analyses. The expression of Bmi1 in s‐OSCC and p‐OSCC was detected by immunohistochemistry (IHC). Radiation‐induced Bmi1 alteration in early‐stage was explored in a rat model and HaCaT cells. After matching, 116 pairs of patients with highly balanced characteristics were included. In univariate analysis, the overall survival (OS), disease‐specific survival (DSS), and local recurrence‐free survival (LRFS) were poorer in s‐OSCC than in p‐OSCC (*P *<* *0.05), while their regional metastasis‐free survival (RMFS) was parallel (*P *=* *0.112). Multivariate analysis further revealed that radiotherapy history was an independent risk factor for OS, DSS, and LRFS (*P *<* *0.05). IHC results showed that the positive rate of Bmi1 was higher in s‐OSCC (*P *=* *0.0027). In a rat model of radiotherapy‐induced mucositis, Bmi1 upregulation was observed 8 days after irradiation. Consistently, Bmi1 was upregulated in HaCaT cells 1 h after irradiation, and its upregulation was in accord with X‐ray exposure duration. In conclusion, the prognosis of s‐OSCC is poorer as compared to p‐OSCC, which may be attributed to Bmi1 upregulation.

## Introduction

Nasopharyngeal carcinoma (NPC) is a specific head and neck malignancy which occurs mainly in Southeast Asia, particularly in south China with an age‐standardized incidence of up to 27–44 per 100,000 persons (1998–2002) 1, 2. Radiotherapy is the primary treatment for NPC because of its high radiosensitivity. In recent years, evolution in treatment technique, especially the development of intensity modulated radiotherapy (IMRT), have brought a dramatic improvement in the prognosis of NPC patients 3. However, ionizing radiation causes a series of complications, such as oral mucositis, xerostomia, osteoradionecrosis, and second primary tumors (SPTs) 4. SPTs, one of the severest late sequelae after NPC radiotherapy, have a devastating effect on cancer survivors 5. The 20‐year cumulative incidence rate (CIR) of SPTs after radiotherapy for NPC was up to 5.37% 6. With the highest standardized incidence ratio (SIR), oral cavity was the most common site to develop SPTs after radiotherapy for NPC 6, 7, 8, 9. Furthermore, 83.9% of the SPTs in oral cavity were oral squamous cell carcinoma (OSCC) 10. Therefore, it is highly representative to study the second primary oral squamous cell carcinoma (s‐OSCC) after NPC radiotherapy, which will provide significant implications for clinical treatment and surveillance for SPTs patients.

Currently, it has been proved that development of s‐OSCC severely affects the prognosis of NPC survivors 6, 11, 12, 13. However, it is still controversial whether s‐OSCC patients exhibit poorer outcome when compared with the primary OSCC (p‐OSCC) patients without a history of radiotherapy 11, 14, 15. Moreover, the pathologic basis of s‐OSCC is largely unknown. Ionizing radiation causes DNA strand breaks in oral basal‐epithelial cells, which proliferate rapidly and are vulnerable to radiation damage 16. Bmi1, one of the polycomb group proteins, can be recruited to DNA damage sites to facilitate damage repair after radiation injury 17, 18. In addition, as a marker of cancer stem cells (CSCs), Bmi1 inhibited the tumor suppressor gene *p16* and was reported to be associated with tumor initiation and progression 19, 20, 21, 22. We suppose that Bmi1 may be altered in s‐OSCC and associated with its pathologic characteristics. To address these problems, we enrolled a total of 2,803 consecutive OSCC patients admitted to Sun Yat–sen University Cancer Center between August 1997 and December 2015 in this study. The s‐OSCC patients were identified and further matched with p‐OSCC patients by propensity score matching (PSM) method to balance the baselines and reduce selection bias 23. The clinicopathologic characteristics were summarized, and the prognosis comparison between s‐OSCC and p‐OSCC patients were analyzed. To further explore the underlying pathologic basis of s‐OSCC, immunohistochemistry (IHC) staining of Bmi1 was performed in clinical specimens. Radiation‐induced Bmi1 alteration in early‐stage was also detected in a rat model and HaCaT cells.

## Materials and Methods

### Patients

**Figure 1:**
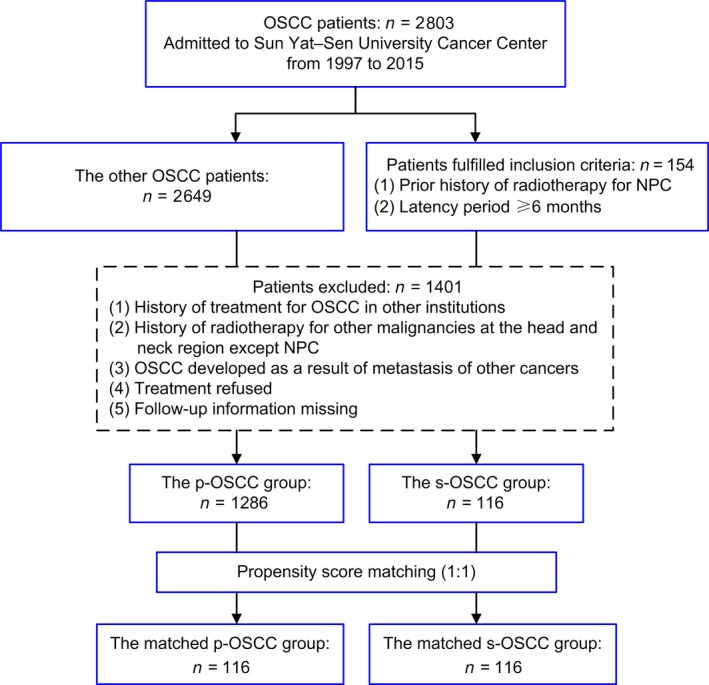
Flowchart of patient selection in this study. OSCC, oral squamous cell carcinoma; NPC, nasopharyngeal carcinoma; p‐OSCC, primary OSCC; s‐OSCC, second primary OSCC after radiotherapy for nasopharyngeal carcinoma.

This retrospective study was approved by the Ethics Committee of Sun Yat‐sen University Cancer Center. Prior informed consent was obtained from patients. A total of 2,803 consecutive patients were admitted to Sun Yat–sen University Cancer Center between August 1997 and December 2015, with pathological diagnosis of OSCC. s‐OSCC patients were identified according to the medical records. The inclusion criteria for s‐OSCC were as follows: (1) a prior history of radiotherapy for NPC, (2) a latency period of at least 6 months between the end of radiotherapy and the diagnosis of OSCC 14, 24. There were 154 patients fulfilled the inclusion criteria for s‐OSCC, and the other 2 649 patients remained in p‐OSCC. The exclusion criteria for all patients were as follows: (1) a history of treatment for OSCC in other institutions, (2) a history of radiotherapy for other malignancies at the head and neck region except NPC, (3) OSCC developed as a result of metastasis of other cancers, (4) treatment refused, (5) follow‐up information missing. Finally, 116 s‐OSCC patients and 1,286 p‐OSCC patients were enrolled for further matching by PSM (Fig. 1).

The clinicopathologic characteristics of the patients were collected. TNM stage of the patients was restaged according to the 7th edition of the AJCC cancer staging manual 25. According to the treatment modalities, patients were classified into three groups 7: curative treatment (surgery or surgery plus concurrent chemoradiotherapy), radiotherapy alone, and chemotherapy alone. The latter two were defined as palliative treatment in the following subgroup analysis.

### Follow‐up

The primary endpoint of this study was overall survival (OS). The second endpoints included disease‐specific survival (DSS), local recurrence‐free survival (LRFS), and regional metastasis‐free survival (RMFS). Please refer to the Appendix S1 for details. The last follow‐up date was 16 June 2016.

### Cell culture and irradiation

The normal human keratinocyte cell line, HaCaT, kindly provided by J. Silvio Gutkind (NIH, Bethesda, MD), was adopted to be irradiated. HaCaT has been tested and authenticated by DNA (STR) profiling. Detailed process is provided in the Appendix S1.

### Animal model

The rat model was modified from our previous work 26. Detailed information is provided in the Appendix S1. All of the animal procedures were conducted in accordance with the Guidelines for the Care and Use of Laboratory Animals and were approved by the Institutional Animal Care and Use Committee at Sun Yat‐sen University.

### Immunohistochemistry (IHC) staining

IHC staining was performed on the rat samples and 50 patient samples available, which included 29 samples in the p‐OSCC group and 21 in the s‐OSCC group. The primary antibodies γ‐H2AX (Abcam, Cambridge, U.K.), 8‐OHdG (Abcam), Bmi1 (R&D Systems, Minneapolis, MN), p16 (Abcam), and p14 (Bioss, Beijing, China) were used. Immunoreactivity in the samples was assessed semi‐quantitatively by calculating the proportion of positive cells according to the reference 27, 28, 29. The experimental information is detailed in the Appendix S1.

### Western blot analysis

The primary antibodies γ‐H2AX, Bmi1, and GAPDH (Cell Signaling Technology, Danvers, MA) were used in Western blot analysis. Densitometry was measured by Image J software (National Institutes of Health, USA). The details are provided in the Appendix S1.

### Statistical analysis

Statistical analysis was performed by SPSS 23.0 software (IBM, Armonk, NY). Detailed information is provided in the Appendix S1. A two‐tailed value of *P *<* *0.05 was considered as statistically significant.

## Results

### Patients and matching

Among all the 2,803 OSCC patients, 5.49% (*n* = 154) of them were developed from NPC patients. For the 116 s‐OSCC patients enrolled in this study, the median interval from NPC radiotherapy to OSCC diagnosis was 10 years (range, 1–39 years). The secondary malignancies occurred within 5 years in 28.4% of the patients and within 10 years in 60.3% of them. The median cumulative radiation dose to the nasopharyngeal region and cervical lymph nodes was 70 Gy (range, 54–96 Gy) and 60 Gy (range, 50–70 Gy), respectively. The average age at s‐OSCC diagnosis was 53.78 ± 9.51 years, and males accounted for the majority of the patients (*n* = 92, 79.3%). There were 37 (31.9%) current or former smokers, and 10 (8.6%) current or former drinkers. The most common tumor site was tongue (*n* = 70, 60.3%) and gingiva (*n* = 27, 23.3%). The histologic grade, T classification, N classification, and TNM stage are showed in Table 1. No patient had distant metastasis at diagnosis, so the M classification was not listed. A majority of patients (*n* = 89, 76.7%) received curative treatment, while a small fraction of patients received radiotherapy alone (*n* = 6, 5.2%) or chemotherapy alone (*n* = 21, 18.1%).

**Table 1:** Comparison of baseline characteristics in the matched cohort

Characteristic	p‐OSCC (*n* = 116)	s‐OSCC (*n* = 116)	*P* value
Age	52.93 ± 11.81	53.78 ± 9.51	0.549
Gender (%)			
Male	89 (76.7)	92 (79.3)	0.634
Female	27 (23.3)	24 (20.7)	
Smoking history (%)			
Yes	40 (34.5)	37 (31.9)	0.676
No	76 (65.5)	79 (68.1)	
Drinking history (%)			
Yes	9 (7.8)	10 (8.6)	0.811
No	107 (92.2)	106 (91.4)	
Tumor site (%)			
Tongue	74 (63.8)	70 (60.3)	0.621
Bucca cavioris	4 (3.4)	5 (4.3)	
Gingiva	20 (17.2)	27 (23.3)	
Palate	18 (15.5)	14 (12.1)	
Histologic grade (%)			
Well	61 (52.6)	64 (55.2)	0.924
Moderate	40 (34.5)	38 (32.8)	
Poor	15 (12.9)	14 (12.1)	
T classification (%)			
T1	39 (33.6)	38 (32.8)	0.875
T2	44 (37.9)	41 (35.3)	
T3	12 (10.3)	16 (13.8)	
T4	21 (18.1)	21 (18.1)	
N classification (%)			
N0	97 (83.6)	98 (84.5)	0.858
N+	19 (16.4)	18 (15.5)	
TNM stage (%)			
I	35 (30.2)	36 (31.0)	0.807
II	39 (33.6)	33 (28.4)	
III	16 (13.8)	20 (17.2)	
IV	26 (22.4)	27 (23.3)	
Treatment modalities (%)			
Curative treatment	95 (81.9)	89 (76.7)	0.618
Radiotherapy alone	5 (4.3)	6 (5.2)	
Chemotherapy alone	16 (13.8)	21 (18.1)	

OSCC, oral squamous cell carcinoma; p‐OSCC, primary OSCC; s‐OSCC, second primary OSCC after radiotherapy for nasopharyngeal carcinoma.

To balance the baselines of s‐OSCC and p‐OSCC patients and minimize the selection bias of retrospective analysis, PSM analysis was performed. After matching, 116 p‐OSCC patients and 116 s‐OSCC patients were included in this study. As showed in Table 1, the two matched groups had highly balanced characteristics, including age, gender, smoking history, drinking history, tumor site, histologic grade, T classification, N classification, TNM stage, and treatment modality. In the curative treatment group, the proportions of patients who received concurrent radiotherapy were parallel between s‐OSCC (5/116, 4.3%) and p‐OSCC (5/116, 4.3%) (*P *=* *1.000). All subsequent analyses were performed based on the propensity score‐matched cohort.

### Poorer OS, DSS, and LRFS in s‐OSCC patients

**Figure 2:**
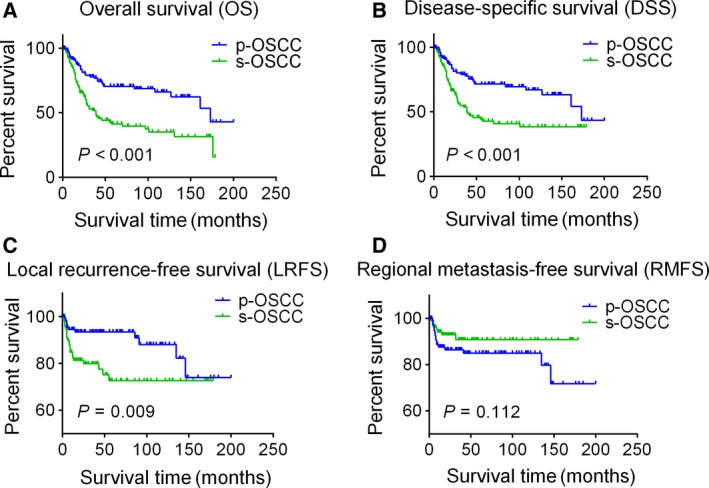
Kaplan–Meier survival curves for s‐OSCC and p‐OSCC patients in the propensity‐matched cohort. (A) Overall survival; (B) disease‐specific survival; (C) local recurrence‐free survival; (D) regional metastasis‐free survival. Survival curves are compared by the log‐rank test, and *P *<* *0.05 is considered as statistically significant. OSCC, oral squamous cell carcinoma; p‐OSCC, primary OSCC; s‐OSCC, second primary OSCC after radiotherapy for nasopharyngeal carcinoma.

In the matched 116 pair patients, the median follow‐up time was 25.0 months (range, 1–179 months) for the s‐OSCC group and 45.5 months (range, 1–200 months) for the p‐OSCC group, respectively. During the follow‐up, 52.6% (61/116) of the patients in the s‐OSCC group and 28.4% (33/116) of the patients in the p‐OSCC group died. The 1‐, 3‐, and 5‐year OS rates were 84.3, 51.7, and 41.2% for s‐OSCC patients and 91.7, 77.8, and 70.4% for p‐OSCC patients, respectively. The OS was significantly poorer in the s‐OSCC group compared with the p‐OSCC group (*P *<* *0.001, Fig. 2A). As it was reported that the poorer survival of s‐OSCC patients might be due to non‐OSCC death 14, the DSS was also analyzed in our study. We found that the DSS was still poorer in the s‐OSCC group than in the p‐OSCC group (5‐year DSS rates, 42.7% vs. 71.3%; *P *<* *0.001, Fig. 2B). During the follow‐up, 19.8% (23 of 116) of the patients in the s‐OSCC group and 9.5% (11 of 116) in the p‐OSCC group developed local recurrence. The LRFS in the s‐OSCC group was significantly poorer than that in the p‐OSCC group (5‐year LRFS rates, 72.7% vs. 93.4%; *P *=* *0.009, Fig. 2C). There were 14.7% (17 of 116) of the patients in the s‐OSCC group and 6.9% (8 of 116) of the patients in the p‐OSCC group developed regional metastasis during follow‐up. However, there was no significant difference in RMFS between the two groups (5‐year RMFS rates, 90.8% for s‐OSCC and 85.0% for p‐OSCC, respectively; *P *=* *0.112, Fig. 2D). Only one patient developed distant metastasis during follow‐up, so distant metastasis was not discussed in this study.

### Radiotherapy history was an independent risk factor for OS, DSS, and LRFS

To identify whether the history of radiotherapy for NPC was an independent risk factor for OSCC patients after adjusted by other variables, univariate and multivariate analyses were adopted. In univariate analysis, radiotherapy history, gender, T classification, N classification, TNM stage, and treatment modality were significantly associated with OS. These variables were then tested in multivariate analysis, and the result showed that radiotherapy history, gender, T classification, and treatment modality were independent risk factors for OS (Table 2). Compared with p‐OSCC patients, s‐OSCC patients took a 2.512‐fold higher risk to die (HR = 2.512, 95% CI 1.636–3.857, *P *<* *0.001). In addition, radiotherapy history was also an independent risk factor for DSS in multivariate analysis (HR = 2.300, 95% CI 1.502–3.65, *P *<* *0.001).

**Table 2:** Univariate and multivariate analyses of risk factors for OS, DSS, and LRFS in the matched cohort

Characteristic	OS			DSS			LRFS		
	Univariate analysis (*P* value)	Multivariate analysis		Univariate analysis (*P* value)	Multivariate analysis		Univariate analysis (*P* value)	Multivariate analysis	
		HR (95% CI)	*P* value		HR (95% CI)	*P* value		HR (95% CI)	*P* value
Radiotherapy history No Yes	<0.001	2.512 (1.636–3.857)	<0.001	<0.001	2.330 (1.502–3.650)	<0.001	0.009	2.756 (1.335–5.688)	0.006
Age (years) <50 ≥50	0.063	–	–	0.068	–	–	0.028	0.430 (0.217–0.852)	0.016
Gender Male Female	0.010	2.620 (1.442–4.762)	0.002	0.012	2.681 (1.440–4.989)	0.002	0.601	–	–
Smoking history No Yes	0.237	–	–	0.225	–	–	0.791	–	–
Drinking history No Yes	0.456	–	–	0.663	–	–	0.824	–	–
Tumor site Tongue Bucca cavioris Gingiva Hard palate	0.179	–	–	0.214	–	–	0.204	–	–
Histologic grade Well Moderate Poor	0.904	–	–	0.668	–	–	0.375	–	–
T classification T1/2 T3/4	<0.001	1.553 (1.244–1.939)	<0.001	<0.001	1.608 (1.281–2.019)	<0.001	0.740	–	–
N classification N0 N+	0.026	–	–	0.015	–	–	0.626	–	–
TNM stage I/II III/IV	<0.001	–	–	<0.001	–	–	0.829	–	–
Treatment modalities Curative treatment Radiotherapy alone Chemotherapy alone	<0.001	1.705 (1.319–2.203)	<0.001	<0.001	1.745 (1.346–2.262)	<0.001	0.253	–	–

OS, overall survival; DSS, disease‐specific survival; LRFS, local recurrence‐free survival; HR, hazard ratio; CI, confidence interval.

As for LRFS, the univariate and multivariate analysis revealed that radiotherapy history and age were independent risk factors for local recurrence (Table 2). Compared with p‐OSCC patients, s‐OSCC patients took a 2.756‐fold higher risk to relapse (HR = 2.756, 95% CI 1.335–5.688, *P *=* *0.006). Consistent with the result of survival curve comparison, radiotherapy history was not an independent risk factor for RMFS in multivariate analysis (data not showed).

### Subgroup heterogeneity of the survival comparison

**Figure 3:**
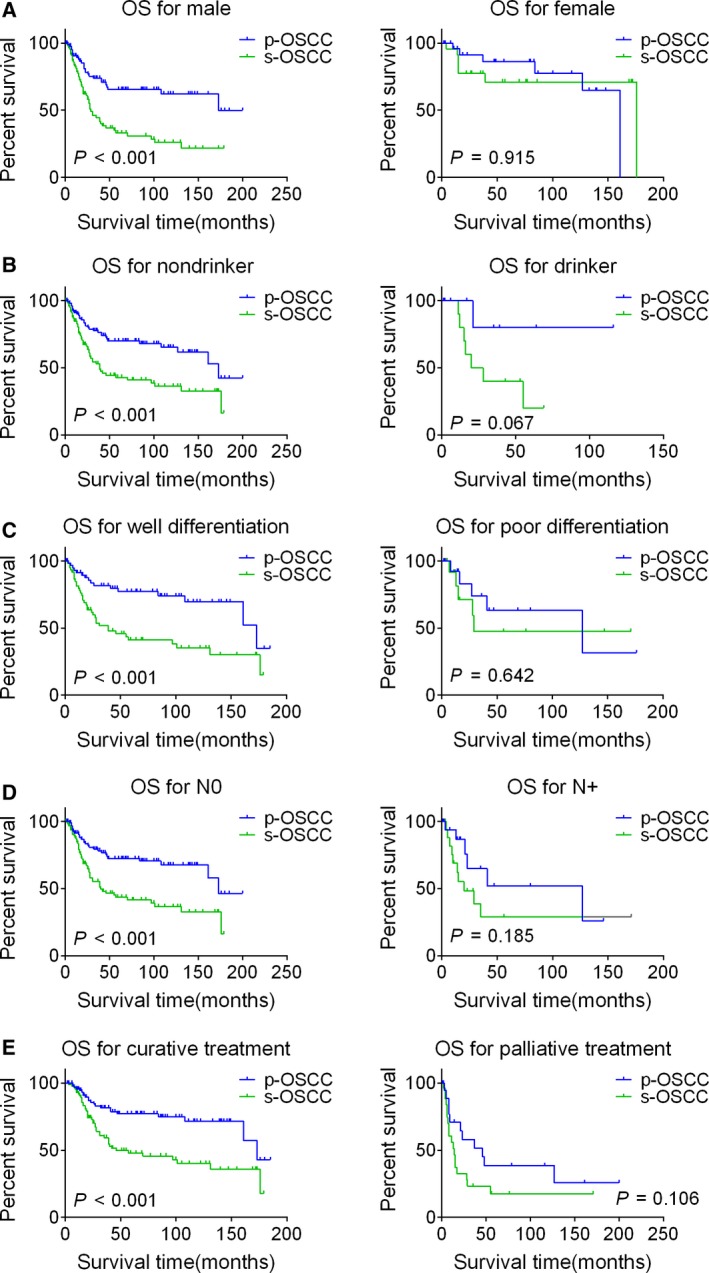
Kaplan–Meier OS curves for s‐OSCC and p‐OSCC patients in the subgroups stratified by gender (A), drinking history (B), histologic grade (C), N classification (D), and treatment modalities (E). Survival curves are compared by the log‐rank test, and *P *<* *0.05 is considered as statistically significant. OSCC, oral squamous cell carcinoma; p‐OSCC, primary OSCC; s‐OSCC, second primary OSCC after radiotherapy for nasopharyngeal carcinoma; OS, overall survival.

**Figure 4:**
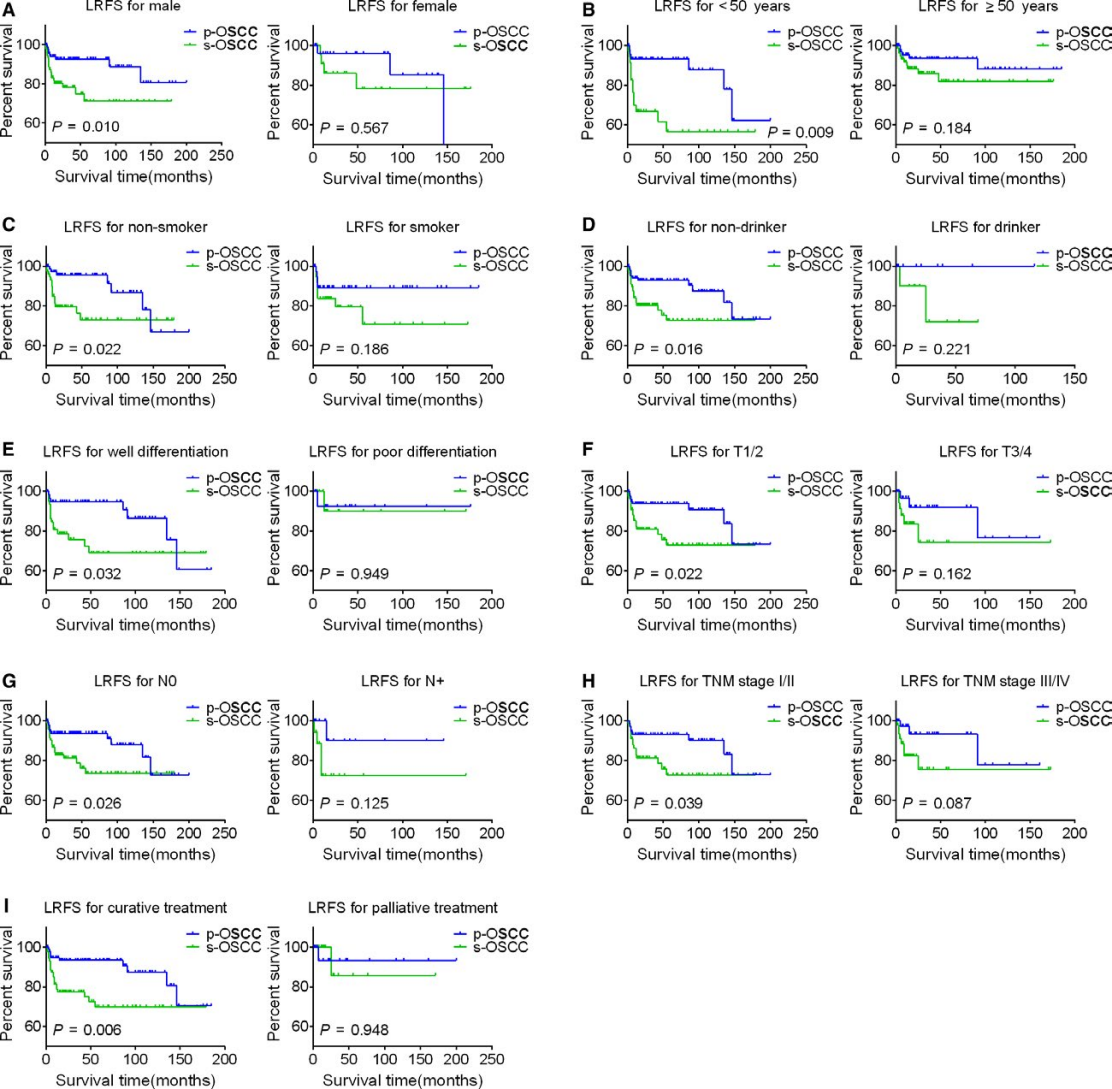
Kaplan–Meier LRFS curves for s‐OSCC and p‐OSCC patients in the subgroups stratified by gender (A), age (B), smoking history (C), drinking history (D), histologic grade (E), T classification (F), N classification (G), TNM stage (H), and treatment modalities (I). Survival curves are compared by the log‐rank test, and *P *<* *0.05 is considered as statistically significant. OSCC, oral squamous cell carcinoma; p‐OSCC, primary OSCC; s‐OSCC, second primary OSCC after radiotherapy for nasopharyngeal carcinoma; LRFS, local recurrence‐free survival.

In order to get more details about the survival discrepancy between s‐OSCC and p‐OSCC patients, subgroup analysis was performed. In the subgroups of male, nondrinker, well differentiation, N0, and curative treatment, s‐OSCC patients exhibited poorer OS than that of p‐OSCC patients, while in the subgroups of female, drinker, poor differentiation, N+, and palliative treatment, the OS was parallel between two groups (Fig. 3). Similarly, heterogeneous results were found when we focused on LRFS. In the male, <50 years, nonsmoker, nondrinker, well differentiation, T1/2, N0, TNM stage I/II, and curative treatment subgroups, s‐OSCC patients showed poorer LRFS compared with p‐OSCC patients; however, in the subgroups of female, ≥50 years, smoker, drinker, poor differentiation, T3/4, N+, TNM stage III/IV, and palliative treatment, the LRFS was comparable between two groups (Fig. 4). For RMFS, the difference remained insignificant between s‐OSCC and p‐OSCC patients in subgroup analysis.

### Upregulation of Bmi1 in s‐OSCC

**Figure 5:**
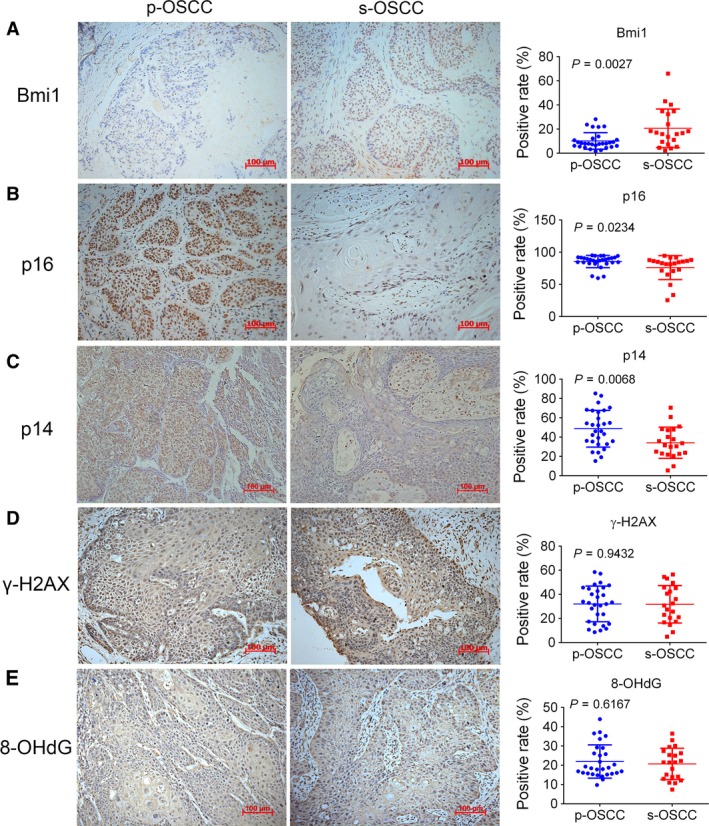
Representative images (200×) of IHC staining for Bmi1, p16, p14, γ‐H2AX, and 8‐OHdG in s‐OSCC and p‐OSCC. *P* values are computed by Student's *t*‐test, and *P *<* *0.05 is considered as statistically significant. Scale bar: 100 μm. IHC, immunohistochemistry; OSCC, oral squamous cell carcinoma; p‐OSCC, primary OSCC; s‐OSCC, second primary OSCC after radiotherapy for nasopharyngeal carcinoma.

To detect the underlying molecular basis of s‐OSCC, IHC staining was performed. The positive rate of Bmi1 was higher in s‐OSCC (20.71 ± 15.62%) than in p‐OSCC (10.22 ± 6.75%) (*P *=* *0.0027, Fig. 5A). As a major downstream target of Bmi1, p16 expression was lower in s‐OSCC (76.14 ± 18.23%) compared with p‐OSCC (85.57 ± 9.30%) (*P *=* *0.0234, Fig. 5B). However, the DNA damage marker, γ‐H2AX, had no significant difference between the two groups (31.79 ± 15.20% in s‐OSCC and 32.11 ± 14.53% in p‐OSCC, *P *=* *0.9432, Fig. 5D). To further verify our results, another commonly used DNA damage marker, 8‐OHdG, was detected. Consistently, results showed that the positive rates of 8‐OHdG were parallel between s‐OSCC (20.73 ± 7.91%) and p‐OSCC (21.94 ± 8.47%) (*P *=* *0.6167, Fig. 5E). As another downstream target of Bmi1, p14 expression was also lower in s‐OSCC (34.16 ± 15.71%) than in p‐OSCC (48.67 ± 18.75%) (*P *=* *0.0068, Fig. 5C).

### Radiation‐induced Bmi1 upregulation in early‐stage

**Figure 6:**
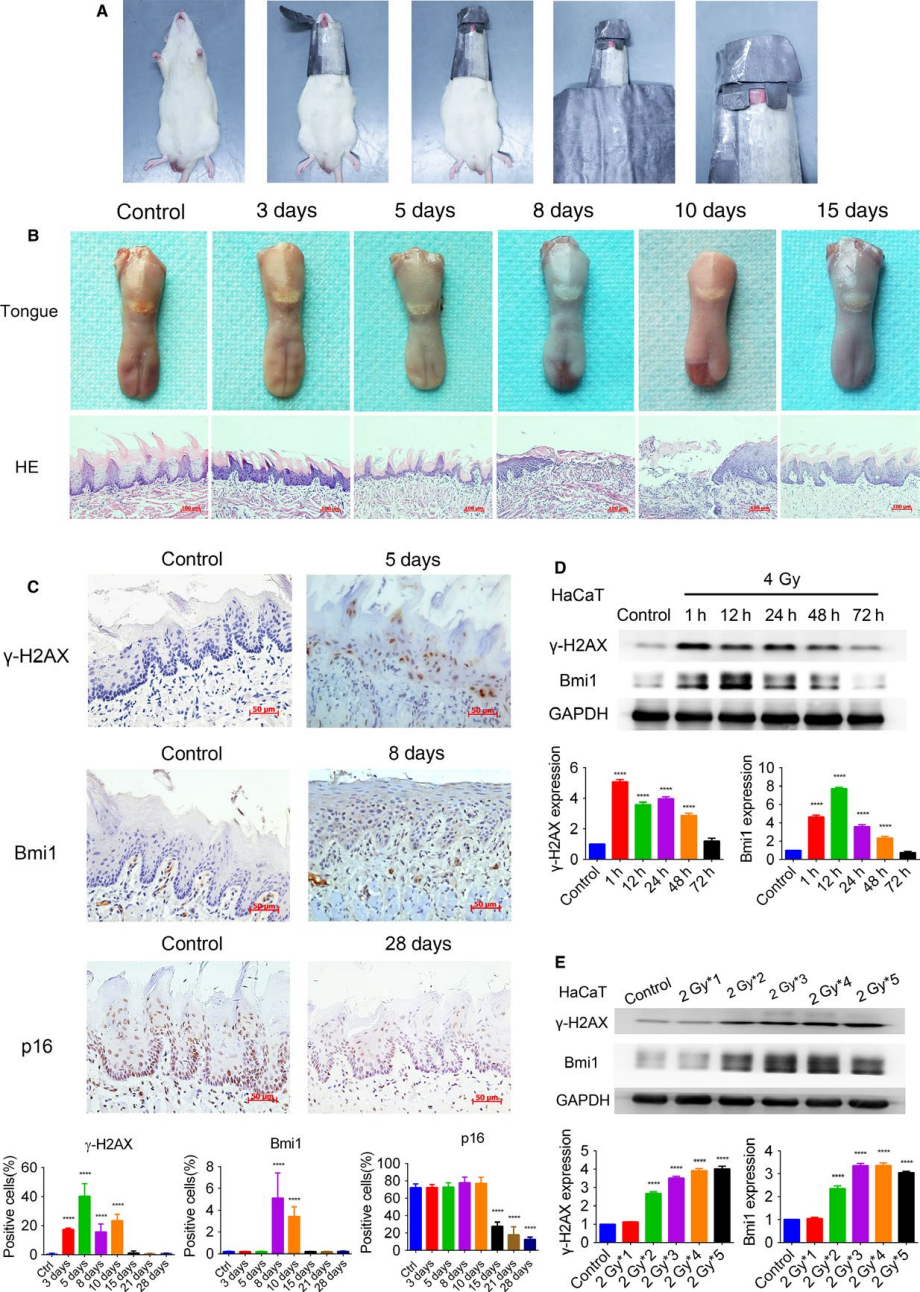
Early‐stage alteration induced by radiotherapy in a rat model and HaCaT cells. (A) Establishment of the rat radiotherapy model. (B) Macroscope and HE staining (200×) of the anterior tongue after irradiated with 25 Gy X‐rays. Latent stage, gradual ulceration stage, and healing stage were observed. Scale bar: 100 μm. (C) Representative images (400×) of IHC staining for γ‐H2AX, Bmi1, and p16 in the anterior tongue after irradiation. The positive rates are compared by one‐way ANOVA and displayed in histograms. Scale bar: 50 μm. (D, E) Western blot analysis demonstrating the alteration of γ‐H2AX and Bmi1 in HaCaT cells after irradiation. The densitometry results are presented as means ± SD and compared by one‐way ANOVA. ****P <* *0.0001 vs. control. HE, Hematoxylin‐Eosin; IHC, immunohistochemistry.

Considering the IHC results of clinical specimens can only indicate long‐term changes after tumor formation, we designed studies to illustrate the molecular alterations soon after radiotherapy. A rat model was established to imitate radiotherapy‐induced oral mucosa injury (Fig. 6A). Macroscopical and histological analysis showed that the irradiated anterior tongue experienced latent stage, gradual ulceration stage, and healing stage after irradiation (Fig. 6B), which was consistent with the typical appearance and progression of radiotherapy‐induced mucositis in clinic. In IHC analysis, the positive rate of γ‐H2AX was significantly increased 3 days after irradiation and reverted to baseline at the 15th day. Bmi1 was upregulated at the 8th and 10th day, whereas p16 was downregulated at the 15th day and persists until the 28th day (Fig. 6C). Furthermore, the normal human keratinocyte cell line, HaCaT, was adopted to detect the alterations in vitro. Western blot analysis revealed that γ‐H2AX and Bmi1 expression in HaCaT cells was upregulated after irradiation (Fig. 6D). In order to imitate fractionated radiotherapy in clinic, HaCaT cells were irradiated with 2 Gy at every cell passage for five times. Importantly, results showed that Bmi1 upregulation was consistent with X‐ray exposure duration (Fig. 6E). In a word, these results revealed that Bmi1 was upregulated and sustained high expression after radiotherapy.

## Discussion

In this study, we compared the prognosis between s‐OSCC patients and p‐OSCC patients and found that s‐OSCC patients had poorer OS, DSS, and LRFS. Meanwhile, we demonstrated that Bmi1 was upregulated in s‐OSCC, as well as in radiation‐induced early‐stage alteration in a rat model and HaCaT cells.

In univariate and multivariate analyses, our results demonstrated that the OS was poorer in s‐OSCC patients when compared with that in the matched p‐OSCC patients. Actually, several previous studies have tried to illustrate their prognostic discrepancy, but the results are contradictory 11, 14, 15. We suspect that the inconsistency may be due to the selection bias and unbalanced baseline characteristics stemming from retrospective studies, which always affect the outcomes and lead to different conclusions. To avoid these drawbacks, a large sample size of 116 s‐OSCC patients were included in our study and PSM method was adopted to imitate randomized control trial 23, 30. Therefore, the influence of confounders and selection bias could be greatly addressed, and we obtained the trustworthy result that the OS was poorer in s‐OSCC patients. Furthermore, to eliminate the influence of non‐OSCC death, the DSS was also analyzed in our study. Similarly, the results also revealed that s‐OSCC patients had a poorer DSS, compared with p‐OSCC patients. Consequently, from our well‐organized study, we could draw a reliable conclusion that s‐OSCC patients have a higher mortality than that of p‐OSCC patients.

In clinic, the high mortality of patients is usually related to the high rate of local recurrence. To learn their connection in our study, we further compared LRFS between s‐OSCC patients and p‐OSCC patients. The results showed that LRFS was poorer in s‐OSCC patients, which was consistent with their high mortality. Except for local recurrence, another cause of treatment failure and poor outcome is always regional lymph node metastasis. However, our results revealed that there was no significant difference for RMFS between s‐OSCC patients and p‐OSCC patients. Previous studies suggested that the lymph node metastasis rate was even lower in s‐OSCC patients 14, 15, 31. This may be the result of lymphatic atresia and altered lymphatic drainage pattern in the neck caused by radiotherapy for NPC 32. In addition, s‐OSCC may have a different biological behavior with decreased metastasis potential 14. These results indicate that the poorer outcome of s‐OSCC patients may be associated with their high rate of local recurrence, rather than regional metastasis.

Furthermore, to investigate the possible subgroup heterogeneity of the results when patients were stratified by clinicopathologic characteristics, subgroup analysis was adopted. Interestingly, we found that the local recurrence rate of s‐OSCC patients was higher than that of p‐OSCC patients among subgroups of T1/2, N0, or TNM stage I/II; however, there was no significant difference when we focused on the subgroups of T3/4, N+, or TNM stage III/IV. Likewise, similar results were also obtained from the comparison of mortality. This finding reminds the clinicians that close cancer surveillance is indispensable for s‐OSCC patients, especially for those in subgroups of T1/2, N0, or TNM stage I/II.

**Figure 7:**
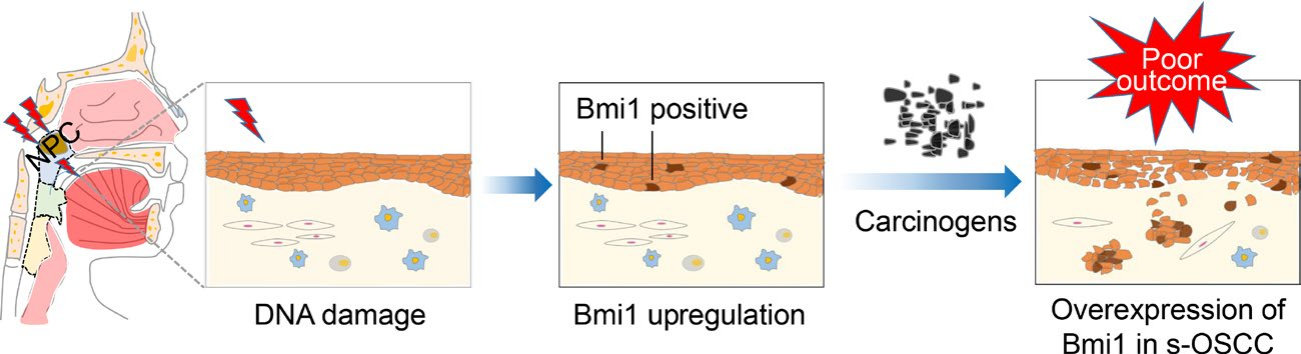
The hypothesis that Bmi1 upregulation contributes to the initiation and progression of s‐OSCC. When the NPC patients treated with radiotherapy, their oral epithelia inevitably receive low‐to‐moderate dose radiation. Radiation injury causes DNA strand break and further upregulates Bmi1 in oral epithelia. Combined with other carcinogens, upregulation of Bmi1 in epithelia facilitates malignant transformation and finally results in s‐OSCC development. Sustained overexpression of Bmi1 promotes progression of s‐OSCC and leads to its poor outcome. NPC, nasopharyngeal carcinoma; OSCC, oral squamous cell carcinoma; s‐OSCC, second primary OSCC after radiotherapy for NPC.

In order to explore the underlying mechanism of s‐OSCC, several possible biomarkers were examined. Importantly, we found that Bmi1 was upregulated in s‐OSCC, accompanied by downregulation of its downstream target p16. These results indicated long‐term changes after radiation exposure in s‐OSCC; however, the molecular alteration in oral epithelia soon after radiotherapy could not be judged. It is unethical to collect oral tissues before malignant transformation in patients who received radiotherapy for NPC. Therefore, we tried to demonstrate the early‐stage changes after irradiation in a rat model and HaCaT cells. In the rat model of radiotherapy‐induced mucositis, Bmi1 was upregulated after DNA damage occurred. Bmi1 upregulation was also observed in HaCaT cells after irradiation, and its expression was increased persistently with the duration of X‐rays exposure. Upregulation of Bmi1 was suggested to promote malignant transformation and tumorigenesis in a series of organs, including oral cavity, liver, intestine, and lung 33, 34, 35, 36. Bmi1‐positive lingual epithelia could serve as CSCs and were considered to be the origin of tongue cancer 37. Furthermore, overexpression of Bmi1 was reported to be associated with poor survival in OSCC patients 38, 39, 40, and targeting Bmi1 could inhibit tumor growth and overcome chemoresistance 21, 41. Collectively, these findings suggest that sustained upregulation of Bmi1 in oral epithelia after radiotherapy may lead to the development and poor outcome of s‐OSCC. Based on our results and previous studies, we put forward the hypothesis that Bmi1 upregulation contributes to the initiation and progression of s‐OSCC. When the NPC patients treated with radiotherapy, their oral epithelia inevitably receive low‐to‐moderate dose radiation. Radiation injury causes DNA strand break and further upregulates Bmi1 in oral epithelia. Combined with other carcinogens, upregulation of Bmi1 in epithelia facilitates malignant transformation and finally results in s‐OSCC development. Sustained high expression of Bmi1 promotes progression of s‐OSCC and leads to its poor outcome (Fig. 7). Further studies are needed to investigate the detailed molecular mechanisms and help to understand the role of Bmi1 in the pathologic process of s‐OSCC.

## Conclusion

In conclusion, this propensity‐matched study revealed that the s‐OSCC was an entity with poorer outcome when compared with the p‐OSCC, especially in T1/2, N0, or TNM stage I/II subgroups. Upregulation of Bmi1 may contribute to the pathogenesis and poor prognosis of s‐OSCC. These data will provide important insights regarding patient surveillance and support further studies for s‐OSCC, as well as for other SPTs.

## Conflict of Interest

None declared.

## Supporting information


**Figure S1.** The absence of p16 in HaCaT cells. Western blot analysis showed that there was no band of p16 in HaCaT cells while striking bands were found in HeLa cells, the positive control of p16.
**Appendix S1.** Methods
