# Supplementary material for: The poor outcome of second primary oral squamous cell carcinoma is attributed to Bmi1 upregulation
Source: Cancer Med. 2018 Feb 26;7(4):1056–69. doi: 10.1002/cam4.1348 (PMC5911571; doi:10.1002/cam4.1348)
Supplement: Supplementary file 1 — Figure S1. The absence of p16 in HaCaT cells. Western blot analysis showed that there was no band of p16 in HaCaT cells while striking bands were found in HeLa cells, the positive control of p16. Appendix S1. Methods [file CAM4-7-1056-s001.pdf]

Supplemental results

Figure S1

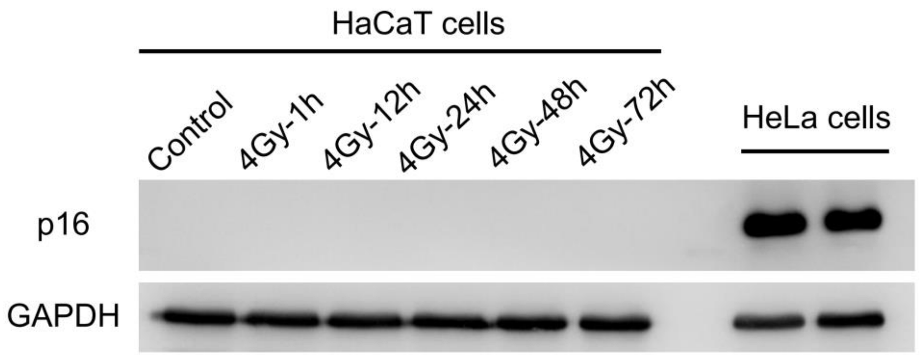

Figure S1. The absence of p16 in HaCaT cells. Western blot analysis showed that there was no band of p16 in HaCaT cells while striking bands were found in HeLa cells, the positive control of p16.

Supplemental methods

Follow up

OS was calculated from the date of OSCC diagnosis until death or the last follow-up date. DSS was defined as the time from OSCC diagnosis to death due to OSCC or complications or last follow-up. LRFS was defined as the time from OSCC diagnosis to local recurrence or last follow-up. RMFS was defined as the time from OSCC diagnosis to regional lymph node metastasis or last follow-up. At each follow-up visit, patients were assessed by physical examination, as well as magnetic resonance imaging (MRI) scan or biopsy when necessary, to detect tumor recurrence or metastasis.

Cell culture and irradiation

The HaCaT cells were maintained in Dulbecco's Modified Eagle Medium (DMEM) (GIBCO,

Grand Island, NY, USA) with 10% fetal bovine serum (FBS; GIBCO) and incubated in a humidified incubator containing 5% CO_2 at 37 ℃. When the cells reached desired density, they were irradiated by an X-ray generator (Rs2000, Rad Source, USA) with a single dose of 4 Gy (160KV, 25mA, 1.28 Gy/min). Cells were harvested 1, 12, 24, 48 and 72 hours after irradiation for western blot analysis. In fractionated irradiation study, cells were irradiated with 2 Gy (1.28 Gy/min) and then harvested or passaged 12 hours after treatment. The process was repeated for 5 times at every cell passage.

Animal model

Forty specific pathogen-free (SPF) male Sprague-Dawley (SD) rats, aged 8 to 10 weeks and weighing 300 to 400 g, were purchased from Jinan Pengyue Experimental Animal Breeding Co. Ltd. Before X-ray irradiation, rats received intraperitoneal injection of 6 ml/kg of a 5% chloral hydrate solution. Except for the anterior dorsal of the tongue, the rest of the animal's body was shielded from radiation exposure (**Figure 6A**). Then, the rats in experimental groups were irradiated with a single dose of 25 Gy X-rays (2.89 Gy/min). At 3, 5, 8, 10, 15, 21 and 28 days after irradiation, 5 rats were sacrificed randomly at each time point via cervical dislocation. Tongue specimens were obtained, fixed in 10% formalin, paraffin embedded and excised for hematoxylin and eosin staining.

Immunohistochemistry (IHC) staining

Paraffin-embedded sections were deparaffinized in xylene and rehydrated in a graded alcohol series. Hydrogen peroxide (3%, 10 minutes) was used to block endogenous peroxidase. Antigen retrieval was applied by heating in citrate buffer (pH 6.0). The slides were incubated with primary antibodies γ-H2AX (Abcam, Cambridge, UK), 8-OHdG (Abcam, Cambridge, UK), Bmi1 (R&D Systems, Minneapolis, MN,

USA), p16 (Abcam, Cambridge, UK) and p14 (Bioss, Beijing, China) overnight at 4˚C. After washed with PBST, slides were incubated with secondary antibody/HRP (GK500705, Gene Tech, Shanghai, China) for 30 min at room temperature and diaminobenzidine (DAB) was used as a chromogen. Nuclear counterstaining was performed with hematoxylin. Then, slides were dehydrated through graded alcohols, cleared in xylene and coverslipped. In negative controls, the primary antibody was replaced by PBS.

Immunoreactivity in the samples was assessed semi-quantitatively by calculating the proportion of positive cells according to the reference [27-29]. Only nuclear staining was considered positive. For each section examined, cells in five randomly selected fields were counted. The assessment was performed by two pathologists blinded from the clinical data.

Western blot analysis

Cells were washed with ice-cold PBS for 3 times and then lysed with RIPA buffer (Sigma-Aldrich, Santa Clara, CA, USA) for 30 minutes at 4 ℃. The protein levels of the lysates were measured using a BCA protein assay kit (Sigma-Aldrich, Santa Clara, CA, USA). Then, the proteins were separated by 10% SDS-PAGE and transferred to a PVDF membrane (Millipore, MA, USA). The membrane was blocked in 5% non-fat milk for 1 hour at room temperature and then incubated with primary antibodies γ-H2AX, Bmi1 and GAPDH (Cell Signaling Technology, Danvers, MA, USA) overnight at 4 ℃. Subsequently, the membrane was washed with TBST for 3 times and incubated with HRP-conjugated secondary antibody for 1 hour at room temperature. The bands were visualized with the enhanced chemiluminescence (ECL) detection system (Millipore, MA, USA) and densitometry was measured by Image J software. Similar results were obtained in three independent experiments.

Statistical analysis

Statistical analyses were performed by SPSS 23.0 software (IBM, Armonk, NY, USA). Logistic regression model was used to develop a propensity score for each patient based on the covariates including age, gender, smoking history, drinking history, tumor site, histologic grade, T classification, N classification, TNM stage, and treatment modality. A 1-to-1 matching (without replacement) by propensity score was performed with the match tolerance 0.1. Continuous variables were compared by Student's t-test and categorical variables were compared by χ^2 test or Fisher's exact test. Survival curves were plotted by the Kaplan–Meier method and compared by the log-rank test. The associations of variables with survival were tested using univariate and multivariate analyses. Univariate analyses were performed using log-rank test and significant variables were further tested in multivariate analyses. Multivariate analyses by Cox proportional hazard model were used to estimate hazard ratios (HRs) and 95% confidence intervals (CIs). IHC results were presented as the means ± SD and compared by Student's t-test or one-way ANOVA. In all analyses, a two-tailed value of $P<0.05$ was considered as statistically significant.
